# Machine Learning-Based probabilistic prediction of glacial lake formation using erosional and topographic features

**DOI:** 10.1038/s41598-025-17401-7

**Published:** 2025-11-05

**Authors:** Anushka Vashistha, Ajay Dashora, Afroz Ahmad Shah

**Affiliations:** 1 Department of Civil Engineering, Indian Institute of Technology Guwahati, Assam, 781039 India; 2 Geosciences Programme, Universiti of Brunei Darussalam (UBD), Bandar Seri Begawan, 110057 Brunei

**Keywords:** Early warning, GLOF, Eastern Himalaya, Erosional features, Google Earth images, Hydrology, Natural hazards

## Abstract

Glacial lake formation in high mountain regions, particularly the Himalayas, is accelerating due to climate-driven glacier retreat, increasing the risk of glacial lake outburst floods (GLOFs) that threaten downstream populations and infrastructure. While climate governs meltwater availability, the formation and evolution of glacial lakes are primarily controlled by geomorphological features such as cirques, valleys, flow channels, retreating glaciers, and neighbouring lakes. However, most predictive models overlook these controls, limiting hazard forecasting capabilities. This study develops a probabilistic framework to predict the probability of glacial lake formation (PGLF) in the Eastern Himalaya by integrating key erosional and topographic features. Using Google Earth imagery and digital elevation models within a 3 × 3 neighbourhood grid structure, we evaluated three predictive models: Logistic Regression (LR), Artificial Neural Network (ANN), and Bayesian Neural Network (BNN). BNN outperformed LR and ANN with an AUC of 0.878, while also estimating both aleatoric and epistemic uncertainties (10⁻³ to 10⁻⁴), enhancing prediction confidence. Neighbouring lakes, cirques, gentle slopes, and retreating glaciers emerged as the most influential predictors, demonstrating the importance of geomorphology, which is often omitted from prior models. The proposed approach offers a transferable framework for identifying high-risk glacial lake formation sites, supporting regional hazard mitigation, early warning systems, and sustainable water resource management in the Himalaya and other glaciated regions. Future improvements should integrate moraine development chronologies, automate data preparation, and incorporate field validation to further refine predictive accuracy and inform global mountain hazard management efforts.

## Introduction

The accelerated retreat of glaciers, largely due to climate change, is contributing to the rapid formation and expansion of glacial lakes, particularly in high-mountain regions such as the Himalayas^1–4^. Observations indicate that smaller lakes expand at higher rates compared to larger ones, reflecting complex feedback between glacial melt, geomorphology, and hydrology. The increasing prevalence of glacial lakes poses significant hazards, as evidenced by the growing frequency of glacial lake outburst floods (GLOFs) in the Himalayas over recent decades^5^. Notably, the Eastern Himalaya exhibits the highest concentration of such events, with GLOF frequency reported to be nearly three times higher than in other Himalayan sectors^6^.

GLOFs are responsible for considerable socio-economic and environmental damage, including loss of life, destruction of agricultural land, damage to hydropower infrastructure, transportation networks, and disruption of downstream ecosystems^2,7–10^. Historical events such as the 1985 Dig Tsho GLOF in Nepal^11^ and the 2012 Kedarnath disaster in India^12^ demonstrate the scale of devastation associated with these events, often affecting communities located tens of kilometres downstream. Given the increasing exposure of vulnerable populations and critical infrastructure, predicting glacial lake formation is essential for effective hazard mitigation, water resource management, and regional development planning.

Existing research has primarily emphasized the role of climate drivers—such as temperature rise, precipitation changes, and glacier mass loss—in shaping glacial lake evolution^2,6^. However, climatic factors influence meltwater production but do not fully explain the spatial distribution of lake formation. Instead, glacial lake genesis is strongly conditioned by the geomorphological and topographical characteristics of the glaciated terrain, including erosional and depositional features such as cirques, valleys, flow channels, moraines, and retreating glacier fronts and tectonics^13^. Depressions formed through glacial erosion, particularly within cirques and valleys, provide the physical space for meltwater to accumulate, leading to the formation of lakes such as tarns and ribbon lakes. Moreover, the interplay of slope, elevation, and terrain curvature governs meltwater routing, lake connectivity, and subsequent hydrological evolution.

Empirical observations across morethan 200 lake sites in the Eastern Himalaya confirm that the coupled dynamics of erosional and topographical features—particularly retreating glaciers, cirques, valleys, and flow channels—fundamentally dictate the occurrence and expansion of glacial lakes^13^. Furthermore, these features modulate sediment transport, influencing lake morphology, turbidity, and stability. Despite their central role, erosional and topographic controls remain underrepresented in predictive models of lake formation, limiting the ability to forecast hazardous lake development in high-mountain environments.

Recent efforts to predict glacial lake formation have utilised geomorphological, statistical, or machine learning (ML) approaches, with varying success. Physically based geomorphological methods, such as those employed by Furian, Loibl^14^, identify potential lake sites by reconstructing bedrock depressions beneath retreating glaciers. However, these approaches are constrained by size thresholds (area greater than 0.1 km²) and often exclude smaller, yet hazardous, lakes. Morphological scoring models (e.g., Zhang, Wang^15^ incorporate expert-assigned weights for features such as slope, crevasses, and glacier proximity but are subject to subjective biases and exclude critical geomorphological variables or features such as cirques and flow channels.

ML-based approaches offer improved capacity to capture complex relationships among influencing factors. Mohanty and Maiti^16^ applied logistic regression (LR) models incorporating climate, topography, and glacier features. However, only 50% of the features used were statistically significant (p-value < 0.05) for lake formation. The insignificance of the remaining features could be attributed to factors such as multicollinearity, weak association with the target variable, or limited data variability. Moreover, LR assumes linear relationships that inadequately capture the inherently non-linear and dynamic processes governing lake formation.

Artificial Neural Networks (ANNs) have demonstrated advantages over linear models by effectively capturing non-linear dependencies and complex interactions in glaciological applications, including glacier boundary detection, meltwater modelling, snow cover forecasting, and ice thickness estimation^17–25^. However, both ANN and traditional statistical regression models (e.g. LR in Mohanty and Maiti^16^ study) lack inherent mechanisms to quantify prediction uncertainty or capture correlations among influencing factors, which can lead to biased or unreliable outcomes. In hazard-sensitive scenarios, such as glacial lake formation, this uncertainty may result in either unnecessary resource allocation due to false positives or unanticipated catastrophic events due to false negatives.

Bayesian Neural Networks (BNNs) address these challenges by integrating Bayesian probability principles with the structural advantages of neural networks. BNNs not only address uncertainty inherent in environmental data and predictions but also effectively capture correlations among a broad range of input features in glaciology. Additionally, it uses small amounts of field data combined with Bayesian inference to train a network, which helps prevent overfitting – a common problem with more ‘data-hungry’ models (e.g. ANN^26–28^). Therefore, this study presents a novel, data-driven framework for probabilistic prediction of glacial lake formation in the Eastern Himalaya using erosional and topographic features within a BNN modelling approach. To our knowledge, this represents the first application of BNNs for forecasting glacial lake occurrence, explicitly integrating geomorphological controls alongside established topographic variables. Comparative assessments are conducted using LR, ANN, and BNN models, with predictive performance evaluated through receiver operating characteristic (ROC) curves and uncertainty quantification metrics.

Our results identify critical geomorphological and topographical features influencing glacial lake formation and demonstrate the superior predictive accuracy and uncertainty management capabilities of the BNN approach. The probabilistic risk maps generated herein provide a valuable tool for hazard mitigation, infrastructure planning, and sustainable water resource management in GLOF-prone regions. On a broader scale, this methodology offers a transferable framework for application in other glaciated mountain systems globally, contributing to improved disaster risk reduction, early warning systems, and climate adaptation strategies.

## Study area

The study area is located in the Eastern Himalaya, covering parts of Arunachal Pradesh (India) and adjacent regions of Tibet. It spans from 28°28′N to 31°13′N latitude and 92°55′E to 98°17′E longitude, with an area of approximately 1,17,796 km². The terrain features steep elevation gradients, ranging from 340 to 7,252 m, with glaciers mostly found between 4,000 and 7,600 m above sea level^29^. The region displays typical high-relief, glaciated mountain landscapes shaped by ongoing tectonic uplift and glacial erosion.

**Fig. 1:**
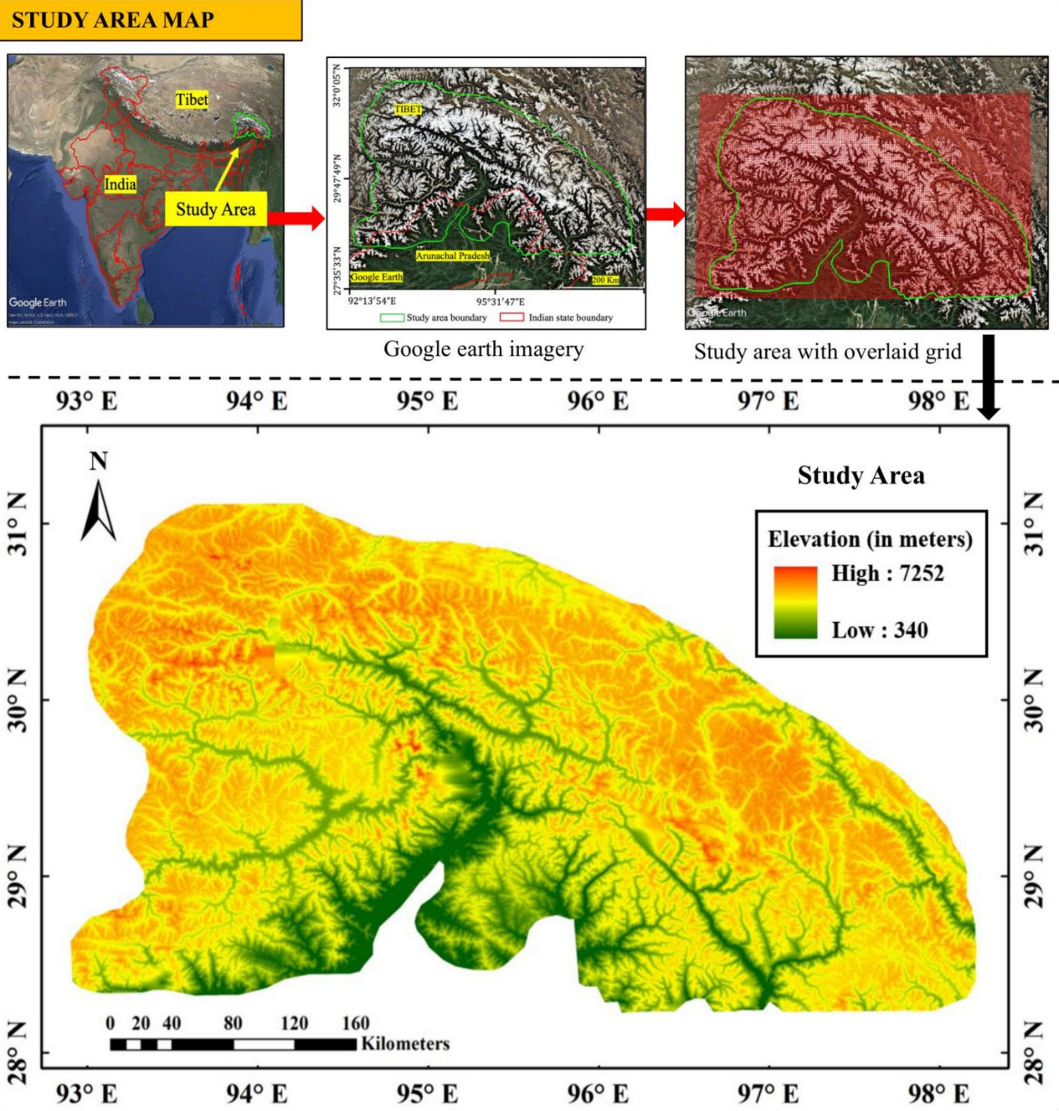
Location and topographic overview of the study area in the Eastern Himalaya, covering parts of Arunachal Pradesh (India) and adjacent regions of Tibet. The top panels illustrate the regional context, the specific study boundary, and the grid overlay used for spatial analysis. The bottom panel shows an SRTM 30 m (DEM) of the study area, with elevation ranging from 340 to 7,252 m. The figure is prepared using Google Earth Pro version 7.3.6.10201 (https://earth.google.com) and ArcMap 10.8 (https://www.esri.com/en-us/arcgis/products/arcgis-desktop/resources). The region exhibits complex mountainous terrain, extensive valley networks, and high-altitude zones where glacier retreat and glacial lake formation processes are active.

The Eastern Himalaya sits at the climatic junction of the Indian Summer Monsoon and the East Asian Monsoon, with transitional zones between these systems strongly influencing the spatial patterns of precipitation, glacier mass balance, and meltwater dynamics^30^. Recent research reports accelerated glacier retreat across the region, resulting in the expansion of existing glacial lakes and the formation of new lakes in erosional depressions^31,32^. The region features a variety of glacial and erosional landforms that change with elevation. Cirques and retreating glaciers are found at higher altitudes, while valleys, flow channels, and glacial lakes are spread across lower elevations and valley floors. Glaciers in the area support perennial river systems that contribute to downstream hydrology, agriculture, hydropower, and community livelihoods, with most rivers eventually draining into the Brahmaputra Basin. Figure 1 shows the location of the study area, along with its elevation profile derived from the 30-meter resolution Shuttle Radar Topography Mission (SRTM) digital elevation model (DEM). The inset panels provide geographic context, highlighting the study area boundary, regional topography, and the spatial grid structure used for data extraction and analysis. The elevation gradient, drainage patterns, and high-altitude zones within the study area represent key controls on glacial processes, lake formation, and downstream hazards.

We collected Google Earth images from the years 2018 to 2022 by overlaying grid over the study area. However, if image scenes captured during the years 2018 to 2022 were obscured by cloud cover or distortion, additional image data from 2015 to 2018 was incorporated to ensure comprehensive coverage. Each image tile within a grid maintained a resolution of 1024 pixels × 768 pixels, corresponding to a size of 3.5 km × 2.63 km (approximately 11m^2^ resolution) on ground. This resulted in a dataset comprising 12,924 images for the entire study area.

## Methodology

This study adopts a data-driven framework to predict the probability of glacial lake formation (PGLF) in the Eastern Himalaya by integrating geomorphological and topographic features within ML models. The workflow comprisesthe following steps:

To begin with, high-resolution Google Earth imagery and SRTM DEM were used to delineate the study area and derive spatial information. The study area was subdivided into a gridded framework to facilitate systematic data extraction. Prediction features were identified across the gridded domain, focusing on key erosional features (cirques, valleys, flow channels, retreating glaciers) and topographic variables (elevation, slope, plan curvature, and profile curvature). Glacial lake presence was also mapped to distinguish between ‘lake’ and ‘no lake’ locations.

A 3 × 3 neighbourhood grid structure was applied to extract feature values for each grid cell associated with either a glacial lake or non-lake location, forming the primary dataset for model development. The dataset was randomly partitioned into training and testing subsets, and class weighting techniques were applied to address data imbalance between lake and no-lake samples. Three ML models, LR, ANN, and BNN were developed to estimate PGLF. Model development incorporated Bayesian optimization of hyperparameters to enhance predictive performance. LR was used to derive feature importance coefficients and p-values, facilitating interpretation of influential predictors. It served as a baseline linear model, while ANN represented a progression toward non-linear modeling, allowing us to assess whether the assumption of non-linearity significantly improves predictive performance of glacial lake formation.

**Fig. 2:**
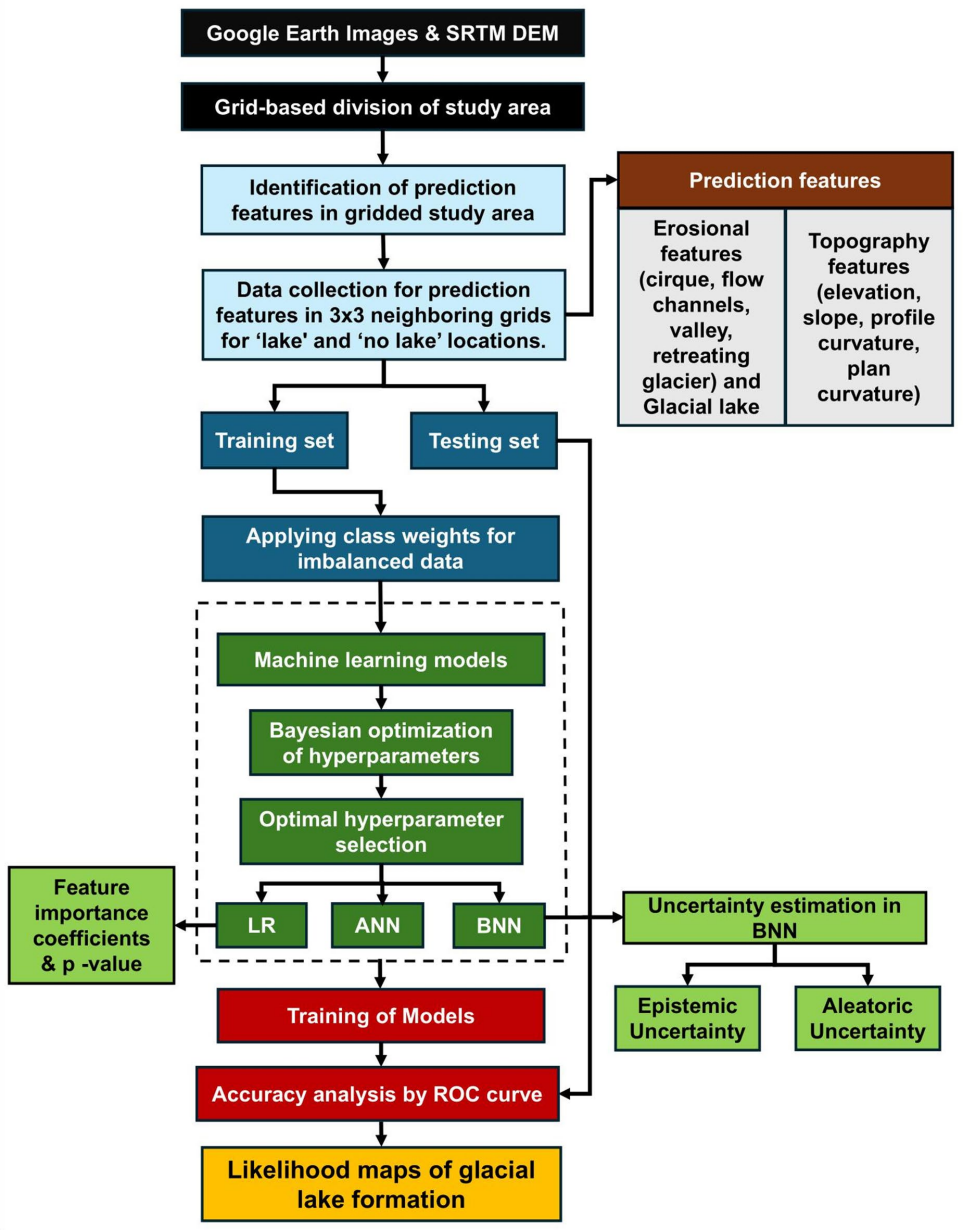
Flowchart depicting the study approach.

BNN was further utilised to estimate predictive uncertainty, capturing both epistemic uncertainty (associated with limited data or model structure) and aleatoric uncertainty (inherent variability in the data). Including both ANN and BNN allowed us to examine the trade-off between deterministic non-linear modelling and probabilistic approaches that explicitly quantify uncertainty. Model performance was evaluated using ROC curve analysis, with area under the curve (AUC) metrics used to compare accuracy across LR, ANN, and BNN approaches. The final output comprises likelihood maps of glacial lake formation across the Eastern Himalaya, providing spatially explicit insights into potential lake development zones and associated uncertainties. These outputs serve as decision-support tools for hazard mitigation, infrastructure planning, and glacial lake monitoring efforts in high mountain regions. The flowchart representing the complete process of obtaining the likelihood maps of glacial lake formation is shown in Fig. 2.

### Data preparation

For the three techniques (LR, ANN, and BNN), input and output data are systematically prepared by measuring the occurrence of erosional features (cirques, valleys, retreating glaciers, flow channels), glacial lakes, and topographical features (elevation, slope, profile curvature, and plan curvature) in each of the 12,924 images. The presence of erosional features and glacial lakes used for dataset preparation is confirmed through multiple visual observations by the authors. Descriptions of erosional prediction features are as follows:

(i) Glacial lakes are uniformly textured water bodies of various sizes and forms, showing a range of colours from deep blue to green or brown. They display differences in depth, sediment content, and sunlight exposure. This study considers all types of lakes, regardless of their dam conditions, such as bedrock-dammed, ice-dammed, moraine-dammed, and other erosion lakes.
(ii) For a glacial lake, a neighbouring lake situated at a higher elevation is defined as an upstream lake, whereas a lake at a lower elevation is considered downstream. However, it is less likely that two adjacent lakes share the same elevation. When lakes are at the same elevation, they form due to the partitioning of inflow at that level. Therefore, in this study, lakes at the same elevation are regarded as part of the downstream lake.
(iii) Cirques are usually identified by a bowl or armchair shape, with a steep headwall, a gently inclined or over-deepened floor, and a convex slope break marking the transition from the cirque to the lower valley.
(iv) Valleys are broad, elongated U-shaped depressions with gently rising slopes as you move upwards, located among hills and often crossed by meandering or braided flow channels.
(v) A retreating glacier is characterised by rough surfaces, crevasses, striations, and the emergence of rocky terrain and moraines, formed by the melting of glaciers and glacio-fluvial erosion.
(vi) Flow channels, which serve as pathways for water across glaciers, can have either a smooth or rough texture depending on whether they are filled with water. Additionally, their colour is affected by sediment load, while their flow pattern (such as braided or meandering) is determined by the surrounding topography.

**Fig. 3:**
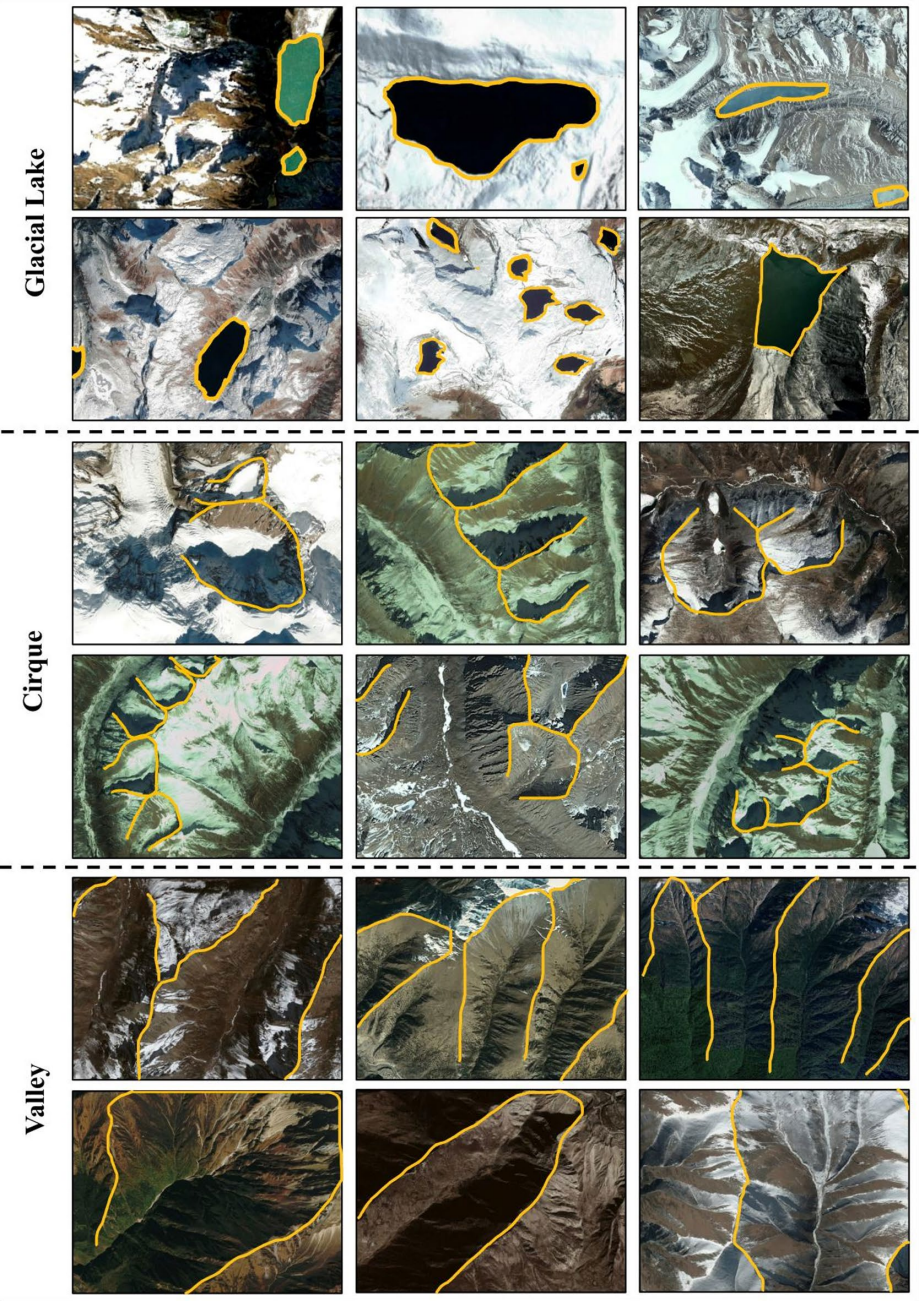
Representative examples of mapped glacial erosional features in the Eastern Himalaya based on high-resolution Google Earth imagery, generated using Google Earth Pro version 7.3.6.10201 (https://earth.google.com). Yellow lines delineate the boundaries of (top) glacial lakes, (middle) cirques, and (bottom) glacial valleys. These features govern the spatial occurrence, expansion, and hydrological connectivity of glacial lakes, influencing downstream flood hazards and landscape evolution. The systematic mapping of these features forms a critical input for the probabilistic prediction of glacial lake formation.

**Fig. 4:**
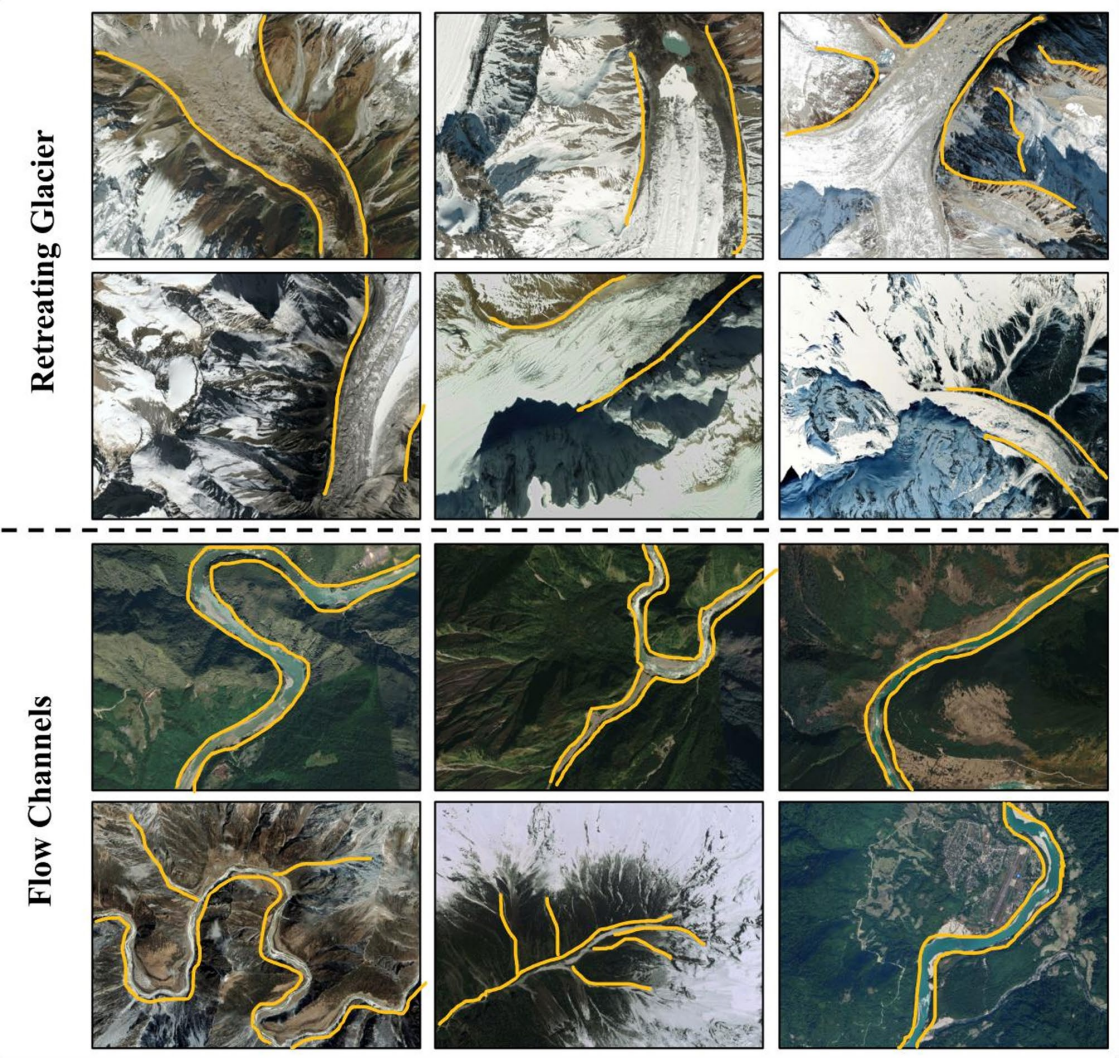
Mapped glacial erosional features in the Eastern Himalaya are shown using high-resolution Google Earth images, generated using Google Earth Pro version 7.3.6.10201 (https://earth.google.com). Visual examples of retreating glaciers (top two rows) and associated flow channels (bottom two rows) in a high mountain region. Yellow lines delineate glacier margins and hydrological flow paths. Retreating glacier images show ice thinning, exposed rock surfaces, and the formation of proglacial lakes, indicative of significant ice loss. Flow channel images illustrate the evolution of drainage networks in response to glacial recession. These visual observations provide contextual support for identifying terrain susceptible to glacial lake formation and hydrological transformation.

Fig. 3 and Fig. 4 show typical examples of prediction features associated with geomorphology, generated using Google Earth Pro version 7.3.6.10201 (64 bit; source: Google Earth Pro).

Further, topography variations influence glacial meltwater dynamics and essentially govern the process of lake formation. Prediction features associated with topography variations like slope, elevation, profile curvature and plan curvatures for each grid are estimated using the global SRTM DEM in ArcMap 10.8 (USGS). Slope influences speed of meltwater flow – steeper slopes cause faster runoff and higher erosion rates, increasing the risk of sediment transport to lower valleys and vice versa. Elevation significantly influences melt rates, as higher elevations retain colder temperatures, slowing the melting of ice. In contrast, lower elevations are warmer, resulting in increased meltwater flow and a higher likelihood of lake formation and downstream impacts. Profile curvature measures the curvature along the direction of maximum slope, which affects how glacial meltwater flows down slopes. Areas with positive profile curvature (upwardly concave, cup shaped or depression) accelerate flow speed, leading to erosion, while areas with negative profile curvature (upwardlyconvex, cap shaped or ridge) decelerate the meltwater movement, encouraging the deposition of sediments. Plan curvature, measuring curvature perpendicular to the slope, affects how meltwater spreads across the terrain. Positive plan curvature (sidewardly convex) areas cause divergent flow, dispersing meltwater and reducing saturation. Conversely, negative plan curvature (sidewardly concave) areas lead to convergent flow, where meltwater collects and increases the potential for ponding or soil saturation. Notably, if the profile or plan curvature is zero, it represents the linear surface along the associated direction^33^. These features collectively help predict water accumulation zones, erosion pathways, and the spread of meltwater, which are vital for assessing flood risks and glacial lake outburst flood (GLOF) hazards in glaciated regions.

### Feature diagnostics and multicollinearity assessment

To ensure uniform scale and stability during model training, all topographic features were standardised to zero mean and unit variance prior to analysis. Simultaneously, erosional features were encoded as binary indicators (presence/absence). Further, the presence of multicollinearity among predictors was assessed using the variance inflation factor (VIF), with results summarised in Table 1.

All features exhibit VIF values well below the conservative threshold of 5, confirming the absence of multicollinearity within the dataset. Specifically, neighbourhood lakes (VIF = 1.54), cirques (1.02), flow channels (1.20), retreating glaciers (1.27), and valleys (1.12) show low VIF values, indicating minimal interdependence while retaining their independent contributions to glacial lake formation modelling. Topographic variables, including elevation (0.51) and slope (0.65), demonstrate particularly low VIFs, reflecting their orthogonality and numerical stability following standardisation. Curvature metrics—profile curvature (VIF = 2.16) and plan curvature (VIF = 2.07)—present the highest VIF values among the predictors but remain within accepted limits, indicating moderate associations with other features without introducing redundancy or instability into the models. These results confirm that all selected features contribute independent, non-redundant information to the modelling framework, validating their inclusion in subsequent ML analyses aimed at predicting the PGLF. The absence of multi-collinearity among features strengthens the interpretability and statistical robustness of the models, ensuring that parameter estimates are stable and free from variance inflation distortions.

**Table 1:** Multicollinearity check using VIF value.

Features	VIF value
Neighbourhood Lakes	1.5400
Cirque	1.0230
Flow channels	1.2040
Retreating glacier	1.2710
Valley	1.1230
Elevation	0.5130
Slope	0.6460
Profile curvature	2.1580
Plan curvature	2.0720

### Logistic regression

Logistic regression (LR) is a multivariate nonlinear statistical analysis method suitable for binary categorical problems with multiple control features expressing relationships between an output or dependent feature and independent (or explanatory) features^34–38^. In this study, presence of lake is output feature or dependent feature (where 1 indicates the presence of a lake in the grid, 0 represents the absence of lake in the grid). Further, independent or explanatory features can be either discrete or/and continuous values depicting the influencing features of lake occurrence. The LR model uses logistic function (also known as sigmoid function), which predicts probabilities values of output feature between 0 and 1. The LR model works by fitting a linear equation to the input features and applying the logistic function to the result of the linear equation. The linear equation defines log-odds ($\:Y$) as:

1 $$\:Y={\beta\:}_{0}+{\beta\:}_{1}{x}_{1}+{\beta\:}_{2}{x}_{2}+\dots\:+{\beta\:}_{n}{x}_{n}$$

Where.

$\:{x}_{1},{x}_{2},\:\dots\:,\:{x}_{n}=\:$Input features in binary (1 for presence of erosional features, else 0) and continuous form (here it is continuous value for topography features).

$\:Y=$ Log odds of probability of outcome (logit function is the natural logarithm of the odds ratio).

$$\:{\beta\:}_{0}=$$ Intercept term.

$\:\beta \:_{1} ,\:\beta \:_{2} , \ldots \:,\:\beta \:_{n} =$ Coefficients of independent features ($\:{x}_{i}$).

The logistic function is applied to the log-odds (determined by Eq. 1) to convert them to probabilities $\:P\left( Y \right)$:

2 $$\:P\left( Y \right) = \:\frac{1}{{1 + e^{{ - Y}} }}$$

where, $\:P$ is the probability of a log-odds of a glacial lake occurrence under the presence of the five erosional features and four topography features.

### Artificial neural network

**Fig. 5:**
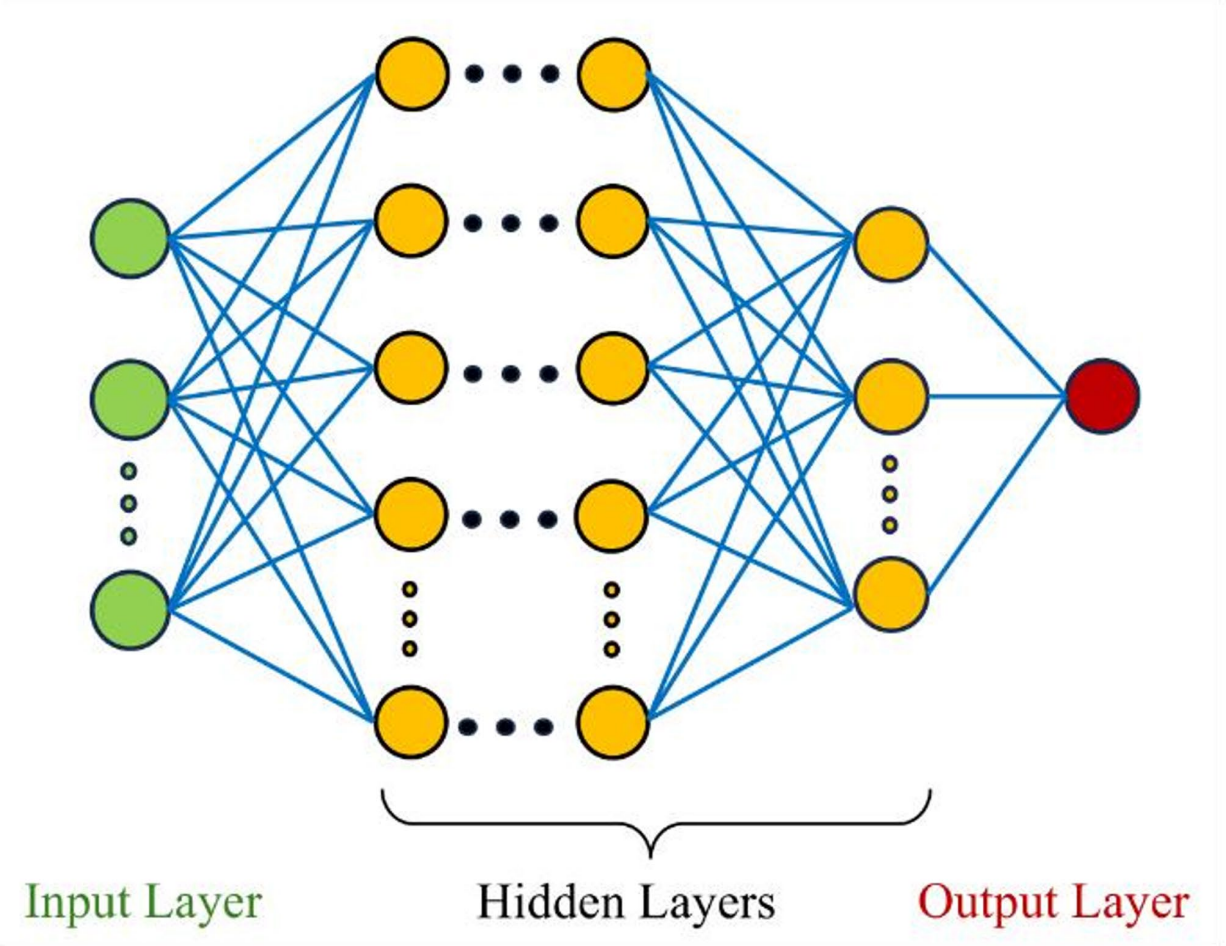
ANN architecture.

An artificial neural network (ANN) is a computational model inspired by structure and function of the human brain^38,39^. Figure 5 shows ANN architecture.

ANNs aim to determine an underlying function linking inputs and output features by processing a large number of data points through weighted connections and activation functions. The data from the layer of input features is transferred from one layer of interconnected nodes (or neurons) to the next, where each neuron processes the received information and passes it further to following layer until final output is generated. To explain an ANN mathematically, consider a dataset $\:{\mathcal{D}} = \left\{ {x_{i} ,y_{i} } \right\}_{{i = 1}}^{N}$, where $\:x_{i} \in \:\mathbb{R}^{d}$ and $\:y_{i} \in \:\left\{ {c_{1} ,c_{2} , \ldots \:,c_{K} } \right\}$. Here, $\:{c}_{k}$ represents one-hot encoded vector, where only the $\:k$-th element is one and the others are zero. Number of different classes is denoted by $\:K$, the sample size by $\:N$, and dimension of input features by $\:d$.

ANNs define model likelihood using a neural network with $\:L$ hidden layers. For each layer $\:j \in \:\left\{ {1,2, \ldots \:,L + 1} \right\}$, let $\:h^{{\left( j \right)}} \in \:\mathbb{R}^{{d_{j} }}$ be the pre-activated output, expressed as:

3 $$\:h^{{\left( j \right)}} = f_{{\theta \:^{{\left( j \right)}} }} \left( {h^{{\left( {j - 1} \right)}} } \right) = W^{{\left( j \right)}} \varphi \:\left( {h^{{\left( {j - 1} \right)}} } \right) + b^{{\left( j \right)}}$$

where, $\:{{W}}^{\left(j\right)}\in\:{\mathbb{R}}^{{d}_{j}\times\:{d}_{j-1}}$ is the weight matrix and $\:{{b}}^{\left(j\right)}\in\:{\mathbb{R}}^{{d}_{j}}$ is the bias vector for the $\:j$-th layer, and $\:\varphi\:\left(\cdot\:\right)$ denotes an element-wise non-linear activation function. By stacking these layers, the output of the ANN is:

4 $$\:f\left( x \right) = f_{{\theta \:^{{\left( {L + 1} \right)}} }} \circ \:f_{{\theta \:^{{\left( L \right)}} }} \circ \: \ldots \: \circ \:f_{{\theta \:^{{\left( 1 \right)}} }} \left( x \right)$$

where, $\:{\theta\:}=\left[\left(\text{v}\text{e}\text{c}\left({{W}}^{\left(1\right)}\right),\dots\:,\text{v}\text{e}\text{c}\left({{W}}^{\left(L+1\right)}\right)\right),\left({{b}}^{\left(1\right)},\dots\:,{{b}}^{\left(L+1\right)}\right)\right]$ represents entire set of network parameters, and $\:vec\left( W \right)$ denotes vectorization of matrix $\:{W}$. Given the final $\:K$-dimensional output $\:f_{{\theta \:}} \left( x \right) = \left( {f_{{\theta \:,1}} \left( x \right), \ldots \:,f_{{\theta \:,K}} \left( x \right)} \right)$, the likelihood $\:p(y|x,\theta \:)$ is written as:

5 $$\:p\left( {y = c_{k} |x,\theta \:} \right) = p(y = c_{k} {\mid }\:f_{{\theta \:}} \left( x \right)) = \frac{{\exp \left( {f_{{\theta \:,k}} \left( x \right)} \right)}}{{\sum \: _{{j = 1}}^{K} \exp \left( {f_{{\theta \:,j}} \left( x \right)} \right)}}$$

During training process, the ANN learns to adjust its parameters $\:{\theta\:}$ by minimizing a chosen loss function, which quantifies difference between predicted outputs of the network and measured target outputs. For classification tasks, cross-entropy loss function is preferred, which is expressed as:

6 $$\:{\mathcal{L}}\left( {\theta \:} \right) = - \sum {\:_{{j = 1}}^{K} } c_{j} \log p\left( {y = c_{j} |x,\theta \:} \right)$$

Training of an ANN involves backpropagation algorithm, which adjusts the network parameters iteratively by propagating error (calculated by loss function) backwards through the network layers. This process enables the network to learn from the error between predicted and labelled outputs to improve its performance over time. The following algorithm expresses functioning of ANN^40^.

**Figure:**
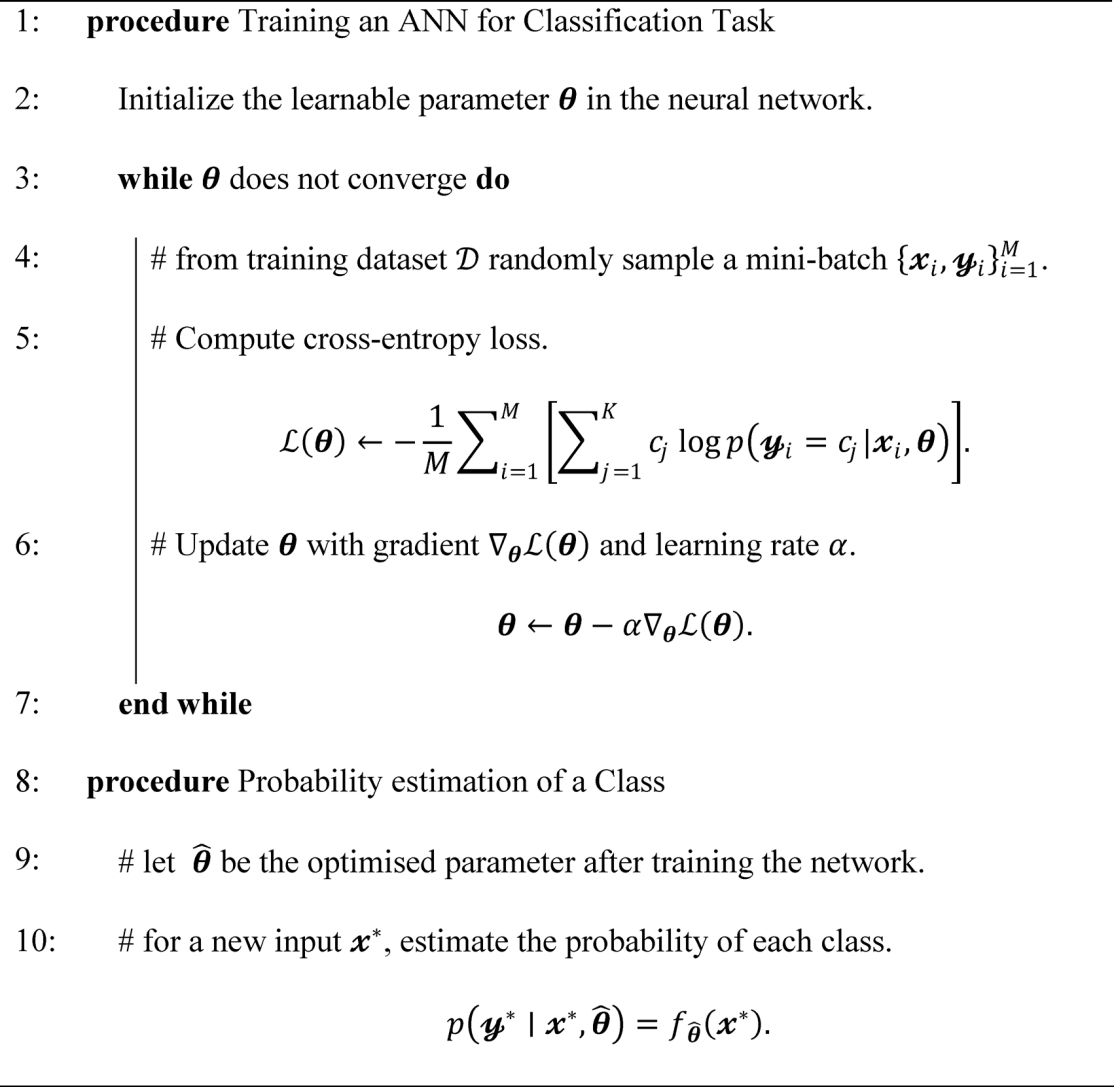
**Algorithm 1 **Training a ANN and probability estimation for a feature class

### Bayesian neural network

Bayesian Neural Network (BNN) is a probabilistic approach to neural networks, offering a framework for quantifying uncertainty in predictions and model parameters. Unlike the conventional neural networks, which provide point estimates for predictions, BNNs provide a distribution of predictions, allowing for more robust decision-making in uncertain environments. Traditional neural networks particularly ANN, frequently encounter overfitting during training due to unbounded weight values and fixed fully connected structures^41,42^. To mitigate this issue and enhance generalization capability, the BNN model is developed for predicting the glacial lake formation ($\:p(y = Lake)$) using the influencing features of lake occurrence as input features ($\:\mathcal{x}$) to the network. Similar to ANN, the BNNs consist of interconnected layers, including input, hidden, and output layers. However, in BNNs, each weight and bias parameter in the network is treated as a random variable with a prior distribution.

During training, BNNs learn the posterior distribution, capturing uncertainty in model parameters as well as uncertainty in data. A prior distribution $\:p\left({\theta\:}\right)$ is placed on the parameter $\:{\theta\:}\in\:{\Theta\:}$ in the Bayesian framework, which computes posterior distribution as:

7 $$\:p\left({\theta\:}|\mathcal{D}\right)=\frac{p\left(\mathcal{D}|{\theta\:}\right)p\left({\theta\:}\right)}{p\left(\mathcal{D}\right)}=\frac{{\prod\:}_{i=1}^{N}p\left({\mathcal{y}}_{i}|{\mathcal{x}}_{i},{\theta\:}\right)p\left({\theta\:}\right)}{p\left(\mathcal{D}\right)},$$

and for given $\:p\left({\theta\:}|\mathcal{D}\right)$ and a new input $\:x^{*}$, predictive distribution for a new output $\:y^{*}$ is given by:

8 $$\:p\left({\mathcal{y}}^{{*}}|{\mathcal{x}}^{{*}},{\theta\:}\right)={\int\:}_{{\Theta\:}}p\left({\mathcal{y}}^{{*}}|{\mathcal{x}}^{*},{\theta\:}\right)p\left({\theta\:}|\mathcal{D}\right)d{\theta\:}.$$

Calculating the posterior $\:p\left({\theta\:}|\mathcal{D}\right)$ is often intractable due to need for integrating over the entire parameter space $\:{\Theta\:}$. To address this challenge, studies employ two primary methods for model learning: Markov Chain Monte Carlo (MCMC) and variational inference. However, MCMC methods proposed by Welling and Teh^43^ and Chen, Fox^44^ require significant memory for training deep networks, making them less practical in certain cases. Variational inference is more attractive as it approximates posterior distribution with a simpler and tractable distribution $\:q_{{\psi \:}} \left( {\theta \:} \right)$, parameterized by $\:{\psi\:}$. It finds the closest distribution to true posterior from a family $\:Q = \left\{ {q_{\Psi } \left( {\theta \:} \right):\psi \: \in \:\Psi } \right\}$, making it computationally feasible for large-scale models. This closeness is measured by the Kullback–Leibler (KL) divergence:

9 $$KL\left( {q_{\Psi } \left( \theta \right)\left. {p(\theta |} \right)~D} \right) = \mathop \smallint \limits_{\Theta } q_{\Psi } \left( \theta \right)\log \frac{{q_{\Psi } \left( \theta \right)}}{{p\left( {\theta |D} \right)}}d\theta .$$

Minimizing the KL divergence equates to minimizing evidence lower bound (ELBO):

10 $${\mathcal{L}}\left( \Psi \right) = - {\mathbb{E}}_{{q_{\Psi } \left( \theta \right)}} \left[ {\log p\left( {y|x,\theta } \right)} \right] + KL\left( {q_{\Psi } \left( \theta \right)\parallel p\left( \theta \right)} \right),$$

where, $\:\mathbb{E}\left[\cdot\:\right]$ is expectation operator.

Method of the variational inference allows optimization by mini-batch learning^28^. The quality of the variational approximation depends on chosen family $\:Q$. While a restricted family aids scalability, it may compromise approximation accuracy^28^.

Recently, Gal and Ghahramani^45^ demonstrated that dropout in neural networks can be interpreted as a form of variational inference in Bayesian neural networks. Similarly^46^, showed that batch normalization in neural networks also aligns with variational inference. These advancements highlight practical applicability of Bayesian neural networks across various settings. The next sub-section discusses methods for quantifying uncertainty in classification tasks using Bayesian neural networks by the variational inference introduced by Gal and Ghahramani^45^ and further developed by Kendall and Gal^47^.

#### Uncertainty quantification in lake prediction using BNN

Variational predictive distribution, which approximates the predictive distribution (posterior probability $\:p\left( {y^{*} |x^{*} ,n{{D}}} \right))$, is defined by Monte Carlo estimator as:

11 $$\hat{p}_{\Psi } \left( {y^{*} {\mid }x^{*} } \right) = \frac{1}{T}\mathop \sum \limits_{{t = 1}}^{T} p_{\psi } \left( {y^{*} {\mid }x^{*} ,\theta _{t} } \right),$$

where $\:{\left\{{{\theta\:}}_{t}\right\}}_{t=1}^{T}$ are samples drawn from optimized variational distribution $\:{q}_{{\psi\:}}\left({\theta\:}\right)$. According to the weak law of large numbers, this estimator converges in probability to $\:q_{{\psi \:}} \left( {y^{*} {\mid }\:x^{*} } \right)$ as the number of samples $\:T$ increases. Once the variational parameters $\:\widehat{{\psi\:}}$ are optimized by minimizing the ELBO, the estimator becomes:

12 $$\hat{p}_{{\hat{\Psi }}} \left( {y^{*} {\mid }x^{*} } \right) = \frac{1}{T}\sum\limits_{{t = 1}}^{T} p \left( {y^{*} {\mid }x^{*} ,\hat{\theta }_{t} } \right),$$

##### Aleatoric and epistemic uncertainties

Optimised network parameter $\hat{\theta }$ is obtained after training a network. Predictive uncertainty $\:Var_{{p\left( {y^{*} {\mid }\:x^{*} ,\widehat{{\theta \:}}} \right)}} \left( {y^{*} } \right)$ can be computed by incorporating negative correlation structures among classes, as proposed by Kwon, Won^48^ as.

13 $$Var_{{p\left( {y^{ * } {\mid }x^{ * } ,\hat{\theta }} \right)}} \left( {y^{ * } } \right) = \underbrace {{\frac{1}{T}\mathop \sum \limits_{{t = 1}}^{T} \left[ {diag\left\{ {p\left( {y^{ * } {\mid }x^{ * } ,\hat{\theta }_{t} } \right)} \right\} - p\left( {y^{ * } {\mid }x^{ * } ,\hat{\theta }_{t} } \right)^{{ \otimes 2}} ~} \right]}}_{{aleatoric}} + \underbrace {{\frac{1}{T}\mathop \sum \limits_{{t = 1}}^{T} \left[ {p\left( {y^{ * } {\mid }x^{ * } ,\hat{\theta }_{t} } \right) - \hat{p}_{{\hat{\Psi }}} \left( {y^{ * } {\mid }x^{ * } } \right)~} \right]^{{ \otimes 2}} }}_{{epistemic}}$$

where, $\:\text{V}\text{a}\text{r}\left[\cdot\:\right]$ is the variance operator, $\:u^{{ \otimes 2}} = uu^{T}$ and $\:diag\left( u \right)$ is the diagonal matrix with elements of $\:\:u$. Predictive uncertainty, $\:Var_{{p\left( {y^{*} {\mid }\:x^{*} ,\theta \:} \right)}} \left( {y^{*} } \right)$ is decomposed into aleatoric (data) uncertainty and epistemic (model) uncertainty (as shown in Eq. 13). Aleatoric uncertainty arises from inherent randomness in the data, while epistemic uncertainty comes from uncertainty in the model parameters^28^. Following is the algorithm for calculations of posterior probability and predictive uncertainty.

**Figure:**
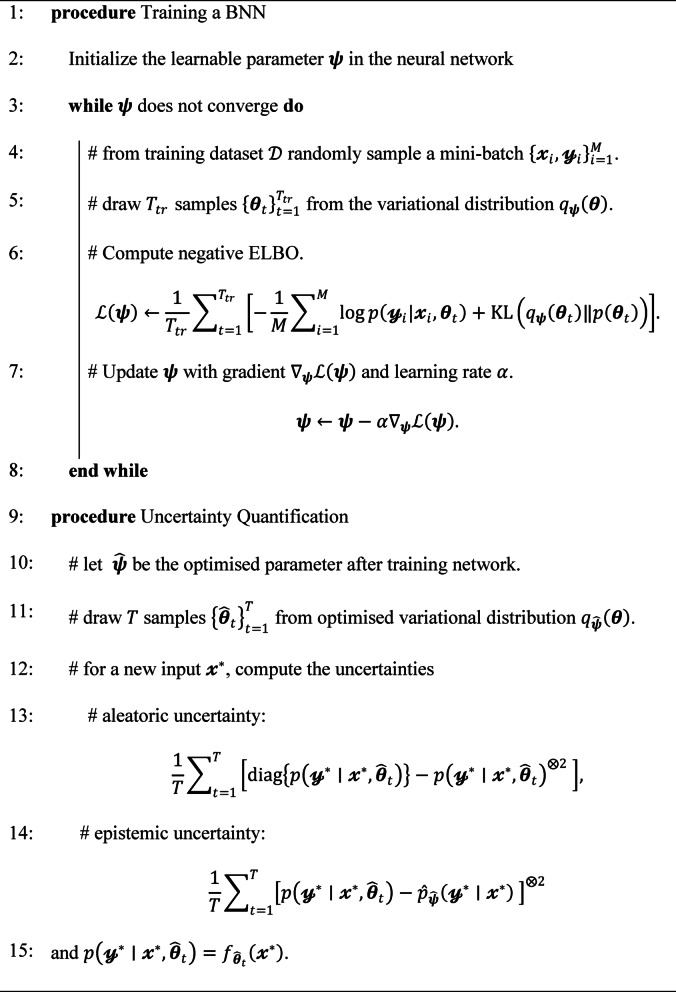
**Algorithm 2** Training BNN and uncertainty quantification with variational inference

### Optimization of hyperparameters

Optimizing hyperparameters is a critical stage in machine learning algorithms, as it directly influences the model’s predictive performance. Configuring the right set of hyperparameters often requires a mix of specialized knowledge, intuition, and trial-and-error experimentation. Classical techniques for hyperparameter optimization include grid search and random search methods. Grid search systematically explores all possible combinations of predefined hyperparameter values to find the best configuration, but it is computationally expensive for large parameter spaces. On the other hand, random search selects random combinations within specified ranges, offering broader exploration and often better results than the grid search method^49^. However, it introduces high variability in results and depends on luck as no strategic sampling is used. Contrary to these two methods, Bayesian optimization identifies the optimal hyperparameters that minimize the validation error. Let $\:X$ represent the space of possible hyperparameters and $\:f$ denote the objective function for minimizing validation error. The optimization process can be expressed as:

14 $$\:{x}^{*}=\text{arg}\underset{x\in\:X}{\text{min}}f\left(x\right),$$

here, $\:{x}^{*}$ represents the set ofhyperparameters that produce the minimum value of the objective function, while $\:x$ refers to any value within the space $\:X$.

This process is classified as a global optimization of black-box functions, where the function expressions and their derivatives are unknown^50^. It utilises a surrogate probabilistic model based on Bayes’ theorem, where the selection of values for subsequent iterations is guided by the outcomes of previous ones^51^. Bayesian optimization is especially effective because the surrogate model is computationally cheaper to evaluate than the actual target function. It strategically narrows down the search space by using prior evaluations, avoiding the inefficiencies of grid search or random search, which often waste time exploring poorly performing areas^52^. This makes Bayesian optimization highly suitable for optimizing expensive or black-box functions, such as deep learning model tuning or scientific simulations. For a more comprehensive understanding, readers may refer to a detailed article by Williams and Rasmussen^53^. There are six stages in Bayesian optimization as follows:

1. Start by evaluating the objective function at a few initial hyperparameter points and construct a surrogate probabilistic model.
2. Use an acquisition function (e.g., Expected Improvement or Upper Confidence Bound) to determine the next hyperparameter set to explore, balancing exploration and exploitation.
3. Evaluate the objective function (e.g., model accuracy or error) for the selected hyperparameters.
4. Update the surrogate model with the new observation, refining its approximation of the objective function.
5. Repeat steps 2–4 for a specified number of iterations or until a stopping criterion is met.
6. Select the hyperparameters with the best-observed performance as the final configuration 

## Simulation

In the study area, 12,924 gridded images are used. For each of the five erosional features, namely, glacial lake, cirque, valley, flow channel and retreating glacier, images in overlaid grid are visualized from bottom row to top row in a column of the overlaid grid. Once information from all images in a column are collected, the same process is repeated for next column till all images in the study area are observed.

Next, for a location, dataset for model training is prepared by observing the occurrence of neighbouring lakes and erosional features within the $\:3\times\:3\:$ neighbouring grids images around central grid. Simultaneously, the dataset for topography features of the central grid is also included for the same location. The central grid image location contains two types of scenarios, either it contains ‘lake’ or it contains ‘no lake’. As few images contain multiple lakes, it is found that 2,462 images locations contain 2,638 lakes, while the remaining 10,462 images show ‘no lake’ occurrence. For neighbourhood lakes, a lake occurring either upstream or downstream with respect to a central grid, plays a unique role in lake formation. Upstream lake act as a reservoir for the lake in the central grid position and provides water through overflow or drainage for the lake formation while downstream lakes indicate established drainage pathways and geomorphic suitability like cirque or valley formations, suggesting favourable conditions for water accumulation in the central grid. Therefore, the input features dataset is prepared by adding information for the presence of neighbourhood lakes, cirque, valley, retreating glaciers and flow channels in neighbourhood of the central grid.

Input features of topography (slope, elevation, profile curvature and plan curvature) associated with ‘lake’ and ‘no lake’ locations are retrieved by using SRTM DEM through ArcGIS. Prepared dataset of the nine features shows information of the surrounding erosional features, and topography variations. The dataset shows an imbalance for the two classes, i.e. 2,462 ‘lake’ and 10,462 ‘no lake’. Out of these 12,924 data locations, 60% data is used to train the three models and 40% for testing. Training with the imbalanced data may result in a biased model that favours the majority class. However, an ideal model should distinguish between both classes accurately. Therefore, to improve the predictive power of the three models, class weights are considered. Although under-sampling is a valid method for class imbalance, it was not used in this study to avoid discarding valuable spatial patterns present in the majority class (no-lake), which are essential for correctly identifying regions unfavourable for lake formation. Afterwards, the trained models are applied to the remaining dataset for computing the probability of lake formation in the entire study area. The following sub-sections discuss the details of optimisation of model hyperparameters and training of the three models.

### Hyperparameters’ optimization

This section discusses the optimization of hyperparameters using Bayesian optimization, including penalty, solver, regularization (C), and maximum iterations for LR models, as well as the number of hidden layers, neurons per layer, epochs, learning rate, activation function, and mini-batch size for ANN and BNN models.

Table 2 shows the option details for LR model hyperparameters and Table 3 presents the varying range of hyperparameters for the ANN and BNN models.

**Table 2:** Description of LR model hyperparameters.

Hyperparameters for optimization	Option details
Penalty type	L1 (lasso), L2 (ridge), elasticnet, none
Solver	saga, liblinear, lbfgs, newton-cg
Regularization (C)	$\:{10}^{-4}-{10}^{4}$
Maximum iterations	$\:50-500$
Class weights	balanced, none

**Table 3:** Range of hyperparameters for tuning the ANN and BNN models.

Hyperparameters for optimization	Range
Number of hidden layer	$\:1-6$
Neurons per hidden layer	$\:10-100$
Epochs	$\:100-500$
Mini batch size	$\:50-150$
Learning rate	$\:0.0001-0.1$
Activation function	Tanh, ReLU, Sigmoid
Class weights	balanced, none

Hyperparameter optimization is conducted by minimizing the cross-entropy loss using *k*-fold cross-validation, where the training data (which corresponds to the complete dataset, excluding the test dataset) is divided into *k* subsets. Each subset is used once as a validation set, while the remaining subsets are used to train the model. This process is repeated until all *k* subsets have been used as the validation set. The average validation error across all *k* folds is calculated and used to identify the optimal hyperparameter combination^54^. In this study, *k* is set to 5, determined through an ablation study that balanced computational cost against accuracy for higher *k* values.

The hyperparameter tuning process begins with initial trials using random combinations of hyperparameters within the defined search space. Following the initial trials, each subsequent iteration involves fitting a Gaussian process and utilizing the posterior distribution, combined with an exploration technique, to identify the next set of hyperparameter combinations to evaluate^50,53^. This process continues until the specified number of trials is completed. Table 4 shows the optimal hyperparameters values for the LR model, while Table 5 presents the optimal hyperparameters for the ANN and BNN models, both selected by minimizing the negative accuracy (maximising accuracy).

**Table 4:** Optimal hyperparameters value for LR model.

Hyperparameters for optimization	Option details
Penalty type	L2 regularization
Solver	lbfgs
Regularization (C)	$\:0.0001$
Maximum iterations	$\:369$
Class weights	balanced

**Table 5:** The optimized values of hyperparameters for ANN and BNN model.

Hyperparameters for optimization	Tuned values for ANN	Tuned values for BNN
Number of hidden layer	$\:3$	$\:3$
Neurons per hidden layer	$\:99$	$\:71$
Epochs	$\:173$	$\:292$
Mini batch size	88	105
Learning rate	$\:0.0021$	$\:0.0026$
Activation function (Hidden layer)	Sigmoid	Sigmoid
Class weights	balanced	balanced

The optimal hyperparameter combinations are then utilized to train the model on the entire training dataset, and the resulting outputs are evaluated on the test set. A detailed discussion of the test results of all the three models (LR, ANN and BNN) is provided in the subsequent section.

### Model simulation

#### Logistic regression (LR) model for lake prediction

The LR model is now trained by using the optimal hyperparameters and coefficients of influencing features are determined with their p-values. Table 6 mentions model coefficients ($\:{\beta\:}_{0}\:to\:{\beta\:}_{9})$ and p-values show statistical significance.

**Table 6:** Coefficients of LR model with p-values.

Input prediction features (Coefficients)	Feature description	Coefficient estimate	*p*-value
Intercept $\:{(\beta\:}_{0})$	not applicable	−2.156	2.55 × 10^−15^
Neighbourhood Lakes $\:{(\beta\:}_{1})$	presence	2.2522	0
Cirque $\:{(\beta\:}_{2})$	presence	0.40431	1.05 × 10^−8^
Flow channels $\:{(\beta\:}_{3})$	presence	−0.12984	0.01427
Retreating glacier $\:{(\beta\:}_{4})$	presence	0.16427	0.00143
Valley $\:{(\beta\:}_{5})$	presence	0.13453	0.02979
Elevation $\:{(\beta\:}_{6})$	metre	−0.00022	7.85 × 10^−5^
Slope $\:{(\beta\:}_{7})$	degree	−0.18355	2.28 × 10^−15^
Profile curvature $\:{(\beta\:}_{8})$	degree per metre	−0.00051	0.01815
Plan curvature $\:{(\beta\:}_{9})$	degree per metre	0.00042	0.08371

So, the equation (Eq. (15) of LR model for the probability of glacial lake formation is as follows:

15 $$\:P\:\left(Glacial\:Lake\right)=\frac{1}{1+{e}^{-\left(Y\right)}},$$

where,

16 $$\:Y=\:-2.156+2.2522{x}_{1}\:+0.40431{x}_{2}\:-0.12984{x}_{3}\:+0.16427{x}_{4}\:+0.13453{x}_{5}\:-0.00022{x}_{6}-0.18355{x}_{7}\:-0.00051{x}_{8}\:+0.00042{x}_{9}.$$

Coefficient values with sign (or beta values) indicate the direction and strengths of the nine predictor features for the lake formation outcome, while p-values assess statistical significance of the features. A p-value less than 0.05 suggests that a feature significantly contributes to the model. Also, negative value of model’s intercept $\:\left({\beta\:}_{0}\right)$ is − 2.156 (with a p-value less than 0.00001), with other predicting coefficients zero, indicates that glacial lake formation is least likely to occur (*P* = 0.1) in the absence of erosional features and appropriate topography.

Among the erosional features contributing positively, neighbourhood lakes have the most substantial positive impact ($\:{\beta\:}_{1}=2.2522$) and p-value equals 0. The proximity of neighbourhood lakes can affect hydrological dynamics by contributing to groundwater seepage or surface flow between neighbouring lakes. This clustering of lakes can create a cumulative effect, whereby lakes collectively influence local water levels and stability, supporting lake formation in nearby basins as upstream lakes play a critical role in transferring water to depressions such as cirques or valleys at lower elevations through flow channels or seepage. Also, overflow or drainage from these upstream lakes can lead to the formation of new lakes in downstream regions. Similarly, the occurrence of downstream lakes indicates established flow paths, suggesting the presence of erosional depressions formed during water inflow. These interconnected hydrological processes emphasize the significant role of neighbourhood lakes in facilitating lake formation.

Compared to neighbouring lakes, cirques, retreating glaciers, and valleys have relatively smaller contributions, as reflected by their coefficient values of 0.40431, 0.16427, and 0.13453, respectively. Nevertheless, their positive coefficients indicate that their presence still increases the likelihood of lake formation. Cirques, with the highest coefficient among the three, provide natural basins that can accumulate meltwater. However, their ability to form lakes depends heavily on water input from external sources, such as upstream lakes. Retreating glaciers contribute meltwater from glaciers, but the rate of production is slower and less concentrated than the substantial reservoir-like releases from upstream lakes.

A small value of the valley coefficient compared to the cirques indicates that the presence of valley landforms has less significance for lake formation. Valleys, shaped as elongated troughs, primarily act as pathways for water flow rather than accumulation, dispersing meltwater and limiting the formation of stagnant water bodies. Moreover, valleys often undergo significant sediment transport and deposition, which can fill potential basins and prevent lake formation. In contrast, sediment deposition in cirques is less impactful due to their steeper slopes and enclosed nature, preserving the over-deepened basin structure for water retention. A study by Mal, Kumar^13^ also highlights that the cirque lakes are more dominant in occurrence than the valley lakes or other erosion-related lakes in the Eastern Himalaya.

On the other hand, a negative coefficient value of flow channels $\:{(\beta\:}_{3}=-0.12984)$ shows that the absence of flow channels will increase the probability of glacial lake formation. This signifies that if flow channels are absent, flow transfers early over a shorter distance to a lake, increasing likelihood of lake formation. On the contrary, if flow channels are present (flow channels are appearing more in numbers and length) it indicates that meltwater will flow for a longer route or pathway, accumulating at downstream regions with substantial delays.

Elevation has a negative coefficient, suggesting that higher elevations decrease the likelihood of lake formation, likely because most of the lakes occur in the elevation range of 4000 m to 5000 m within the study area. Notably, this elevation is below the equilibrium line of altitude, which ranges from 5500 m to 6000 m in the Eastern Himalaya^55^. This also indicates that most of our study area with lakes is in the environment of an ablation zone that promotes the likelihood of lake formation. However, the effect of elevation is minimal due to the small coefficient size.

Slope, with a significant negative coefficient $\:{(\beta\:}_{7}=-0.18355)$, indicates that as the slope increases (steeper slopes), it reduces the likelihood of lake formation as it helps in water drainage rather than retention. Contrarily, flatter areas are more favourable for water accumulation and support lake formation. Profile curvature, with a negative and significant coefficient (p-value = 0.0181), shows that regions with convex surface profiles favour lake formation, as they promote water movement to the depression and retention in one place. Finally, plan curvature (horizontal terrain curvature), have a very small positive coefficient and it is the only features which is statistically insignificant (p-value > 0.05), implying that plan curvature have a minor influence on the likelihood of lake formation in this model.

#### Artificial neural network (ANN)

We have defined ANN architecture with nine features in input layer, followed by three fully connected hidden layers, each with 99 neuronsand sigmoid activation functions to introduce non-linearity. Finally, a fully connected layer contains two neurons corresponding to two classes (lake and non-lake), and the class outputs are converted to class probability values by a softmax function. The output from the softmax layer is passed to the classification layer, where the model is trained by minimizing the cross-entropy loss as given in Eq. (6). Class weights are applied to handle any class imbalance, giving more importance to underrepresented classes in the training process. Training data labels, initially represented by 0 and 1 (for lake and non-lake, respectively) are converted to a one-hot encoded vector. The model is trained using the Adam optimizer for up to 173 epochs with a mini-batch size of 88, and an initial learning rate of 0.0021. Additionally, validation data is provided to monitor the model’s performance throughout the training process.

#### Bayesian neural network (BNN)

In BNN, weights and biases are varied by a Gaussian distribution. $\:\mathcal{N}(\mu\:,\:{\sigma\:}^{2})$, where $\:\mu\:$ represents the mean and $\:{\sigma\:}^{2}$ the variance. During training, the BNN learns the means and the variances of Gaussian distributions, which ultimately determines the distribution of the weights and biases.

We propose using a scale mixture of two Gaussian distributions as the prior, where both distributions have a zero mean but different variances. The mixture distribution is given by:

17 $$\:p\left({\theta\:}\right)=\pi\:\mathcal{N}\left(0,{\sigma\:}_{1}^{2}\right)+\left(1-\pi\:\right)\mathcal{N}\left(0,{\sigma\:}_{2}^{2}\right),$$

where, $\:{\sigma\:}_{1}^{2}$ and $\:{\sigma\:}_{2}^{2}$ are variances of two mixture components. The first component has a higher variance $\:\left({\sigma\:}_{1}>{\sigma\:}_{2}\right)$, leading to a broader prior distribution with a heavier tail. The second component has a small variance $\:\left({\sigma\:}_{2}\ll\:1\right)$, concentrating many of the prior values around zero^56^. The variances are decided by learning during training. The prior distribution follows a Gaussian mixture model with $\:\mu\:=0$, $\:{\sigma\:}_{1}=1$, $\:{\sigma\:}_{2}=0.5$ and mixing proportion $\:\pi\:=0.5$.

The BNN architecture includes a feature input layer with nine units corresponding to the input features. Following this, three Bayesian fully connected layers, each with 71 neurons and Sigmoid activation functions participated. These layers compute average weights and biases of the weight distribution during training. Last Bayesian fully connected layer has two neurons corresponding to the two output classes (lake and non-lake), with a softmax function converting the output into class probabilities.

The BNN is constructed and small values of specific learning rate are applied to ensure the Bayesian parameters remain close to their initial values. An initial learning rate for the priors is set to 0.25 to update the prior probabilities of the weight distributions, while a learning rate of 0.0026 is used to update the means and variances of layer weights and biases. The training is carried out over 292 epochs with a mini-batch size of 105. The Adam optimizer is employed for updating the network parameters, and both the Evidence Lower Bound (ELBO) loss and validation loss are monitored continuously. After training, the developed BNN model is applied to the entire study area, providing a spatial probability map for lake formation. Additionally, uncertainty estimates are generated through posterior sampling, aiding in more informed decision-making for predicting lake formation.

## Results and interpretations

### Mapped glacial erosional features

Mapping of glacial erosional features reveals the dominant role of glacial processes in shaping the landscape and hydrology of the Eastern Himalaya. Key landforms—including glacial lakes, cirques, and U-shaped valleys—were identified through high-resolution satellite imagery (Fig. 3), illustrating their geomorphological significance and their influence on glacial lake formation and evolution. Glacial lakes appear in various morphologies and spatial configurations, typically occupying over-deepened basins formed by glacial erosion or subsequent ice retreat. Their irregular or elongated shapes reflect intense glacial sculpting, and their presence highlights regions of significant ice mass loss and topographic modification. Cirques, depicted in the middle rows of Fig. 3, are steep-walled, amphitheater-like hollows located near the origins of former glaciers. These high-elevation features act as natural accumulation basins for snow and ice, and upon retreat, often give rise to small tarns or nascent glacial lakes. At lower elevations, U-shaped valleys dominate the landscape, as shown in the bottom rows of Fig. 3. These valleys represent the primary conduits for meltwater and sediment transport, linking high-altitude glacierized zones with downstream river systems and lowland catchments. Interpretations drawn from satellite observations also highlight active glacial retreat and related hydrological responses (Fig. 3). Evidence of exposed bedrock, thinning ice tongues, and expanding proglacial lakes underscores the rapid pace of ice loss driven by regional climate warming. The flow channels observed in deglaciated and vegetated terrains—ranging from braided streams to incised river networks—demonstrate how meltwater reconfigures the landscape. In several areas, these channels traverse steep gradients and narrow valleys, signaling the potential for high-energy hydrological events such as debris flows, sediment surges, and GLOFs.

Therefore, the features illustrated in Figs. 3 and 4 emphasize the interdependence of glacial erosion, meltwater hydrology, and terrain evolution in high-mountain regions. The accurate delineation of glacial lakes, cirques, and valleys provides foundational input for predictive models of glacial lake development. Concurrently, visual evidence of glacier retreat and emerging flow channels highlights the transformation of hydrological systems and the growing risks to downstream communities. This integrated understanding is essential for anticipating the impacts of climate-driven glacial change on regional water resources and natural hazards.

### Model performance and accuracy assessment

**Fig. 6:**
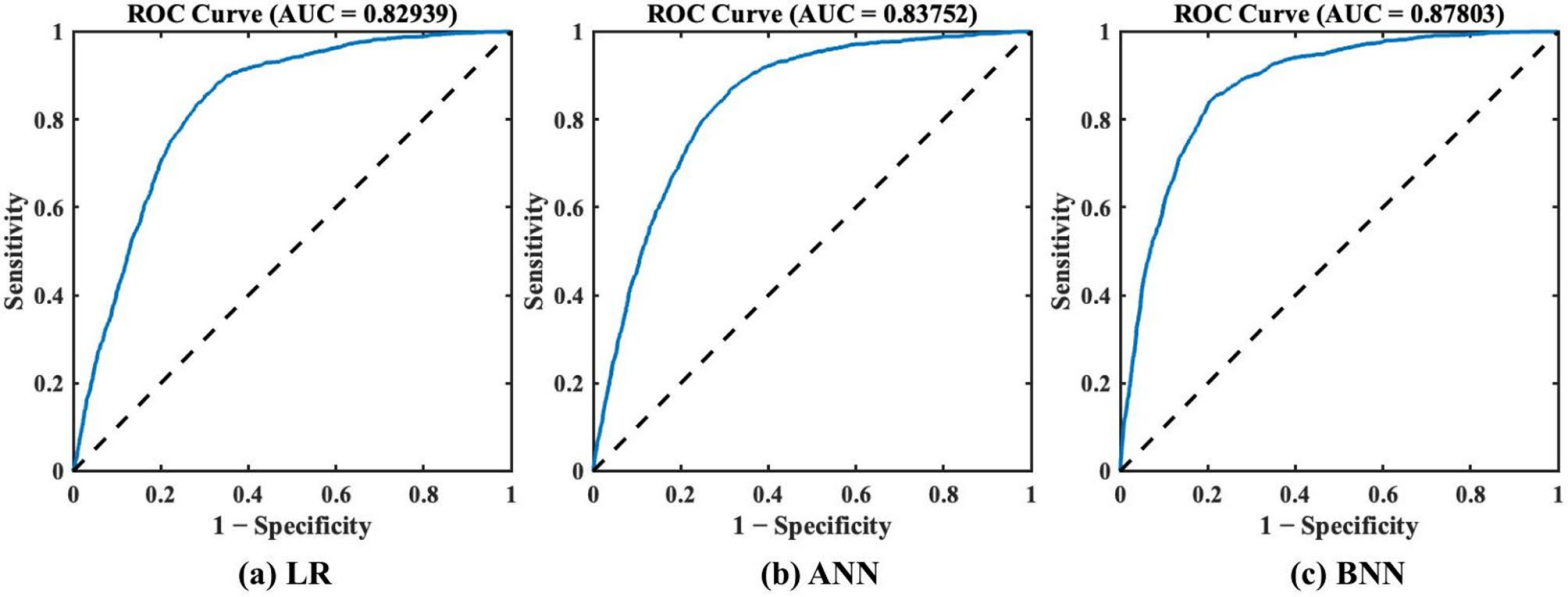
Model accuracy based on ROC curve for the test dataset: (a) LR, (b) ANN, and (c) BNN.

After training the LR, ANN and BNN models using predictor variables, the accuracy analysis of the models is performed by using the ROC curves. Figure 6 presents the ROC curves, which present the performance of the LR, ANN and BNN models in classifying ‘lake’ and ‘no lake’ instances for the test dataset. The ROC curve represents the trade-off between true positive rate (sensitivity) and false positive rate across varying classification thresholds, with the AUC serving as a metric for overall model discrimination. The LR model achieved an AUC of 0.829, indicating moderate capability in distinguishing ‘lake’ from ‘no lake’ locations (Fig. 6a). The ANN model improved upon this result, with an AUC of 0.837, reflecting enhanced ability to capture non-linear relationships between predictor variables (Fig. 6b). The BNN model exhibited the highest classification accuracy, achieving an AUC of 0.878 (Fig. 6c). The ROC curve for the BNN model closely approaches the top-left corner, indicating high sensitivity and a low false-positive rate, both critical attributes for reducing resource expenditure and operational risks during field validation in remote, high-altitude environments. Comparative analysis shows that all models perform substantially above random classification (AUC = 0.5), with the BNN model significantly improving predictive accuracy and reliability over LR and ANN.

### Spatial prediction and uncertainty quantification

**Fig. 7:**
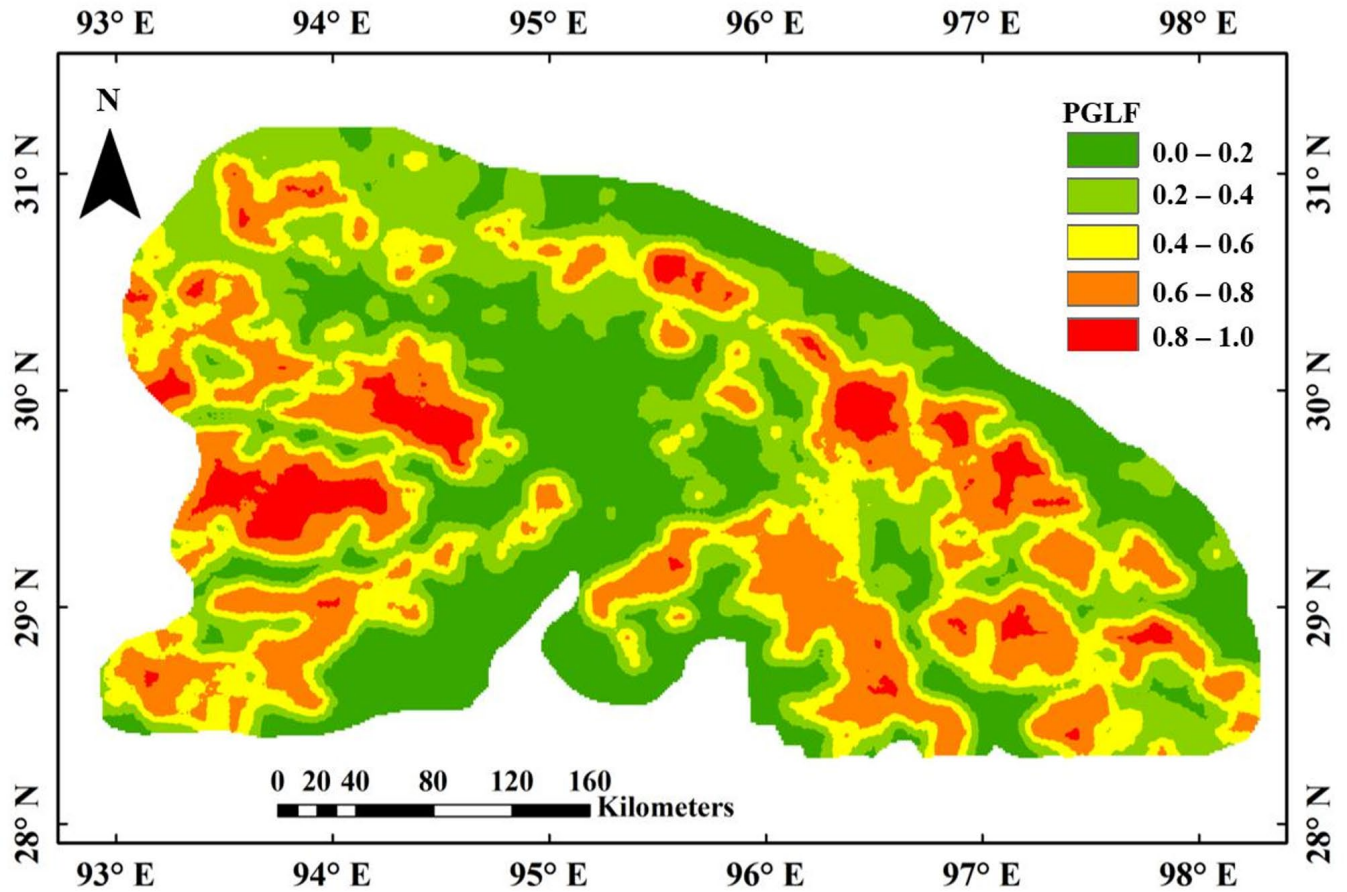
LR model-based probability map of glacial lake formation generated in ArcMap 10.8 software (URL: https://www.esri.com/en-us/arcgis/products/arcgis-desktop/resources). The LR-derived map shows colour-coded probability, where red indicates high and dark green low probabilities of lake formation.

**Fig. 8:**
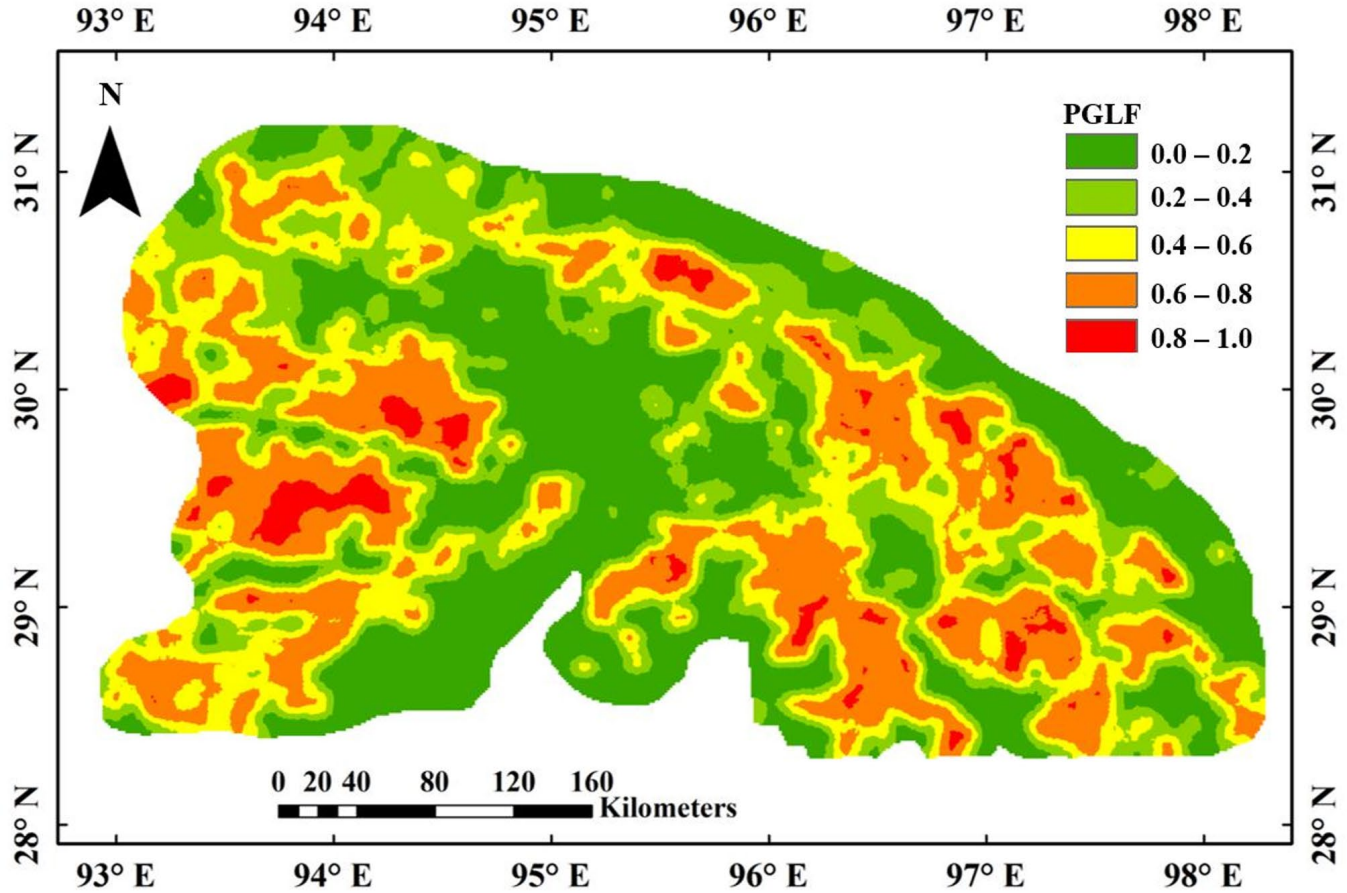
ANN model-based probability map of glacial lake formation generated in ArcMap 10.8 software (URL: https://www.esri.com/en-us/arcgis/products/arcgis-desktop/resources). It displays colour-coded probabilities, where red indicates high and dark green low chances of lake formation.

**Fig. 9:**
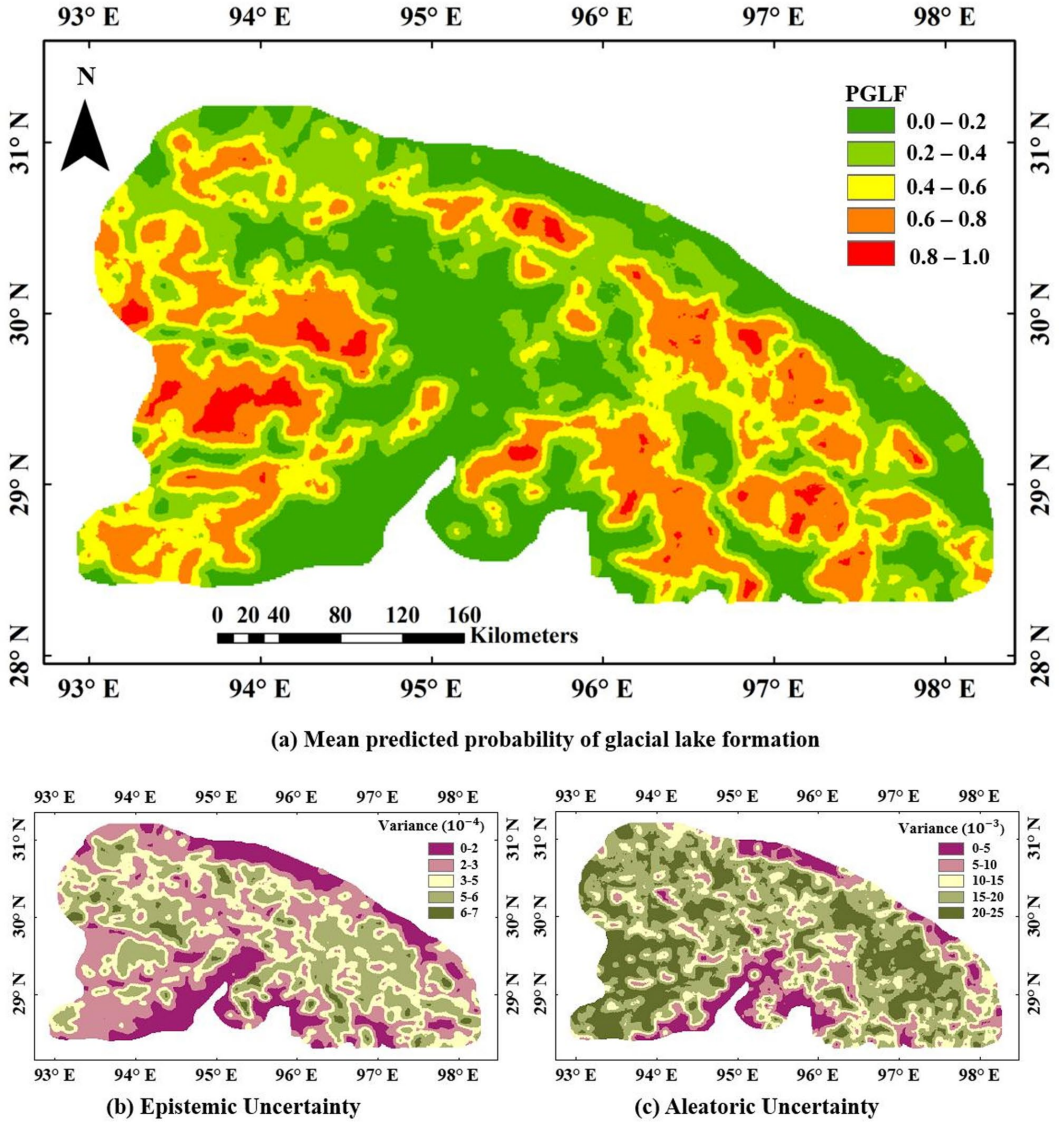
Spatial distribution of the BNN model based on PGLF and its associated variance, generated in ArcMap 10.8 software (URL: https://www.esri.com/en-us/arcgis/products/arcgis-desktop/resources). The top panel shows PGLF values categorised from very low (0.0–0.2, green) to very high (0.8–1.0, red), indicating areas with varying likelihood of glacial lake development. The bottom-left panel depicts variance in PGLF predictions at a scale of 10⁻⁴, while the bottom-right panel shows variance at 10⁻³. Higher variance areas (purple to light brown) indicate greater uncertainty in model predictions and identify zones requiring further investigation.

Continuous probability maps generated using kriging interpolation illustrate the spatial distribution of glacial lake formation likelihood across the Eastern Himalaya (Figs. 7 and 8, and 9). These maps classify probabilities into five distinct ranges: very low (0–0.2), low (0.2–0.4), moderate (0.4–0.6), high (0.6–0.8), and very high (0.8–1.0). Regions of very high probability, delineated in red, correspond to areas with geomorphological conditions conducive to lake formation. Contrarily, very low likelihood regions (0 to 0.2) are marked with green colour, indicating the locations where lakes are least likely to form. These maps provide critical information for identifying existing glacial lakes, predicting regions susceptible to future glacial lake formation, and informing hazard mitigation strategies related to GLOFs. The LR-based probability map (Fig. 7) shows a spatially constrained cluster of high-probability locations, whereas the ANN model (Fig. 8) displays broader, more spatially distributed zones of elevated lake formation likelihood. The BNN model (Fig. 9a) demonstrates the most extensive and spatially coherent representation of very high-probability regions, reflecting its superior ability to resolve complex spatial patterns associated with glacial lake development.

Figure 9 illustrates the spatial distribution and associated uncertainty of the PGLF across the study area. The top panel presents the PGLF values, categorized into five probability classes as mentioned in previous paragraph. High to very high probabilities (orange to red regions) are predominantly found in central, southeastern, and some southwestern zones, indicating favorable conditions for future glacial lake development. These areas are likely influenced by glacial retreat, steep topography, and high meltwater input. In contrast, the northwestern and some central areas show lower PGLF values (green), suggesting limited likelihood of lake formation due to less favorable geomorphological or climatic conditions. The bottom two maps illustrate the variance associated with the PGLF estimates, which reflects the uncertainty in model predictions. The bottom-left map shows epistemic uncertainty with variance $\:{(\sigma\:}^{2})$ on the order of 10⁻⁴, while the bottom-right map represents aleatoric uncertainty with higher magnitude variance on the order of 10⁻³. The areas with higher variance (light brown to purple shades) are mostly located in the southern and southwestern parts of the study region, suggesting increased uncertainty possibly due to limited input data, higher terrain complexity, or model sensitivity in those areas. Regions with lower variance (green to yellow) correspond to zones with more consistent environmental and topographic characteristics, indicating higher model confidence. The total uncertainty of the prediction is estimated by adding the epistemic and aleatoric uncertainty. Estimates of the total uncertainty can be used further for evaluating the upper and lower bounds of the confidence interval. This suggests that the BNN model is the most effective in explaining the complex and non-linear relationship between the erosional features and the lake formation compared to the other two models (LR and ANN).

### Probability distribution and feature influence

**Table 7:** PGLF-based grid distribution for ‘no lake grid locations’ (total grids = 10,462).

Model	PGLF based grid distribution across the probability ranges				
	0.0-0.2 (Very Low)	0.2–0.4 (Low)	0.4–0.6 (Moderate)	0.6–0.8 (High)	0.8-1.0 (Very high)
LR	4853	2207	927	1658	817
ANN	5322	1444	1253	1762	678
BNN	4908	2116	1323	1571	544

**Table 8:** PGLF based grid distribution for ‘existing lake grid locations’ (total grids = 2,462).

Model	PGLF based grid distribution across the probability ranges				
	0.0-0.2 (Very Low)	0.2–0.4 (Low)	0.4–0.6 (Moderate)	0.6–0.8 (High)	0.8-1.0 (Very High)
LR	117	199	286	1049	812
ANN	126	141	310	1093	792
BNN	78	155	187	1017	1025

Analysis of probability distributions for ‘no lake’ (n = 10,462) and ‘lake’ (n = 2,462) grid locations reveals key patterns (Tables 7 and Table 8). In ‘no lake’ locations, all models consistently identify substantial portions of the landscape within the very low (0–0.2) and low (0.2–0.4) probability ranges, with LR predicting 4853 and 2207 locations, ANN identifying 5322 and 1444, and BNN predicting 4908 and 2116, respectively. This indicates that at these locations, the models have predicted a lower chance of likelihood of glacial lake formation, reflecting minimal influence. Notably, 4105 locations in the very low probability range (0.0 to 0.2) are consistently identified by all three models. It is found that neighbouring lakes are not present in all these locations and on steep slopes. In contrast, within moderate probability regions (0.4–0.6), the BNN model predicts 1323 grids, marginally higher than ANN (1253) and LR (927), reflecting increased model sensitivity to transitional conditions where lake formation potential is uncertain. Of particular relevance are the very high probability (0.8–1.0) regions, where 334 locations are consistently identified by all three models within ‘no lake’ areas, suggesting geomorphological conditions highly favourable for future lake formation despite the current absence of surface water bodies. These locations predominantly feature neighbouring lakes and cirques, with varying occurrences of retreating glaciers (280 locations), valleys (286), and flow channels (196), all coinciding with gentle slopes.

For ‘lake locations’ (Table 8), a similar trend is observed. In the very low (0 to 0.2) to low (0.2 to 0.4) probability ranges, LR predicts 117 and 199 locations, ANN identifies 126 and 141, and BNN predicts 78 and 155 locations. The predictions with low probability suggest low confidence in lake formation at these grids and indicate that lake-forming characteristics are less pronounced here. Notably, BNN predicts the least number of existing lake (true positive lake) locations with a very low probability, while LR and ANN predict a higher number. In the moderate probability range (0.4 to 0.6), the number of predicted locations increases, with LR identifying 286, ANN predicting 310, and BNN identifying 187, representing transitional regions with moderate favourability for lake formation. In high range (0.6 to 0.8) and very high range (0.8 to 1.0) of probabilities, LR predicts 1049 and 812 locations, ANN predicts 1093 and 792, and BNN identifies 1017 and 1025, suggesting strong influence from lake-forming features. Further, prediction of higher number of lake locations (true positive lakes locations) by BNN followed by ANN and LR in very high probability range, presents the BNN model’s superior effectiveness and reliability in understanding the non-linear complex influence of all the predictor variables.

Notably, 492 locations in the very high probability range (0.8 to 1.0) are common across all three models, indicating a strong likelihood of lake formation driven by predictor features, particularly erosional features. As LR model indicated dominance of erosional features in lake formation, we further observed the occurrence of influencing features in these locations. It is found that the neighbouring lake and cirques are the most critical features, which are occurring in the neighbourhood of all 492 locations. Further, among these 492 locations, 406 feature valleys, 381 contain retreating glaciers, 304 exhibit flow channels with a gentle slope and varying occurrence of remaining topography features. These findings also highlight the significant role of erosional features, particularly neighbouring lake, cirques, valleys, retreating glaciers, and flow channels in determining lake formation probabilities.

Building upon the earlier comparison of LR, ANN, and BNN models, we further extended our analysis to include additional ML models capable of providing predictive uncertainty, namely, Monte Carlo Dropout and Deep Ensemble Neural Networks. These models estimate uncertainty by introducing stochasticity during inference. However, these methods use heuristic sampling techniques and lack a formal probabilistic framework. In contrast, BNNs incorporate uncertainty directly into the training process via Bayesian inference, allowing for a more principled and calibrated estimation of both epistemic and aleatoric uncertainties. Table 9 illustrates the performance of all models using the AUC metric derived from the ROC analysis.

**Table 9:** Comparison of AUC values (%) for different machine learning models used in predicting glacial lake formation likelihood.

Machine learning models	AUC value (%)
LR	82.93
ANN	83.75
BNN	87.8
Monte Carlo dropout	84.31
Deep ensemble neural networks.	83.89

The results demonstrate that the BNN model achieves the highest AUC value (87.8%), indicating superior predictive accuracy in distinguishing lake-forming from non-lake-forming regions. Although Monte Carlo Dropout (84.31%) and Deep Ensemble models (83.89%) also provide uncertainty estimates and show improved performance over standard ANN (83.75%) and LR (82.93%), they still fall short of the accuracy and reliability offered by the BNN model. This highlights BNN’s advantage as the most robust and informative approach for probabilistic glacial lake formation prediction under uncertainty.

## Discussion

The present study provides the first quantitative demonstration of the predictive value of erosional and topographic features—cirques, valleys, flow channels, retreating glaciers, and neighbouring lakes—for assessing glacial lake formation likelihood in the Eastern Himalaya using a BNN framework. This builds upon, but significantly extends, earlier efforts that relied on either linear statistical approaches (e.g^16^., or subjective geomorphological assessments^14,15^. The limitations of LR models, including assumptions of linear relationships and statistical insignificance of parameters, are evident in the constrained spatial prediction and lower AUC performance observed here. Similarly, while ANNs capture non-linear relationships more effectively, their inability to quantify predictive uncertainty undermines their reliability in operational settings, particularly where hazard management and resource allocation are concerned^23,24^. In contrast, BNNs incorporate both epistemic and aleatoric uncertainty into the prediction process^26,27^, offering not only improved classification accuracy but also critical information on model confidence. This capability is essential for high-altitude hazard assessments, where ground-truth validation is logistically challenging, resource-intensive, and potentially hazardous. Comparison with alternative uncertainty-aware models, including Monte Carlo Dropout and Deep Ensemble Neural Networks, reinforces the advantage of the BNN approach. Although these models improved upon standard ANN performance (AUC of 84.31% and 83.89%, respectively), they remain inferior to BNNs both in predictive accuracy (AUC = 87.8%) and principled uncertainty estimation, which is consistent with previous applications in glaciological hazard forecasting^57,58^. The findings also provide empirical support for the central role of geomorphological controls in glacial lake development. Specifically, the consistent association of neighbouring lakes and cirques with high lake formation probabilities aligns with established theories of glacial erosion, overdeepening, and meltwater accumulation^6,13^. Importantly, while the current framework integrates erosional features effectively, future work should prioritise incorporating depositional features, particularly the chronology of moraine formation and sediment dynamics, to improve model resolution and robustness. Field-based geomorphological surveys and targeted dating of moraine complexes would address existing limitations and refine probabilistic assessments of lake formation and stability. Therefore, this study demonstrates that integrating high-resolution geomorphological data with advanced probabilistic modelling offers a scalable, transferable approach to predicting glacial lake formation under data-sparse, high-uncertainty conditions. The BNN framework provides an essential tool for GLOF risk assessment, early warning system design, and sustainable development planning in the Himalaya and other glaciated mountain systems globally.

## Conclusion

The rapid growth of glacial lakes in the Eastern Himalaya, driven by climate-induced glacier retreat, increases GLOF risk. Predicting lake formation has been limited by poor integration of geomorphological controls. This study creates a probabilistic model including features like cirques, valleys, flow channels, retreating glaciers, nearby lakes, elevation, slope, and curvature to better predict glacial lake formation likelihood. Our comparative analysis of three predictive models, specifically LR, ANN and BNN, demonstrates that BNN provides the most reliable predictions, achieving an AUC of 0.878. This outperforms LR (AUC = 0.829) and ANN (AUC = 0.837), underscoring the advantage of Bayesian frameworks in capturing complex, non-linear processes governing glacial lake formation. Notably, BNN uniquely quantifies both aleatoric (data-driven) and epistemic (model-driven) uncertainties, ranging between 10⁻³ and 10⁻⁴, thereby enhancing forecast reliability, a crucial requirement for hazard mitigation planning in the Himalaya.

Although LR identifies the relative influence of geomorphic predictors, highlighting neighbouring lakes, cirques, slope, and retreating glaciers as key controls, its limited accuracy restricts its practical application. In contrast, BNN successfully identifies 2,042 true-positive glacial lake locations, particularly within zones where neighbouring lakes, cirques, gentle slopes, and active glacial retreat coincide. The spatial convergence of 492 high-probability locations across all three models further validates the geomorphic controls underlying lake formation processes. This work advances glacial hazard forecasting by explicitly integrating geomorphology into a transferable, uncertainty-aware modelling framework. The findings not only support improved monitoring of glaciofluvial erosion and lake development but also inform proactive disaster risk reduction strategies in rapidly evolving mountain environments.

Despite advancements, key limitations persist, especially the resource-intensive need for high-quality, manually prepared data. Future efforts should automate data workflows, include depositional features like moraine chronologies, and add field validation to improve predictions. These upgrades will enhance our capacity to forecast glacial lake development and handle risks amid climate and geomorphological changes.

## Data Availability

Image data used in this study is obtained from Google Earth Pro (https://earth.google.com). Digital Elevation Model (DEM) is obtained from USGS earth explorer website (https://earthexplorer.usgs.gov). Full details of data preparation is provided in methodology Sect. 3. Also, the processed data is available on request to the corresponding editor.
